# Effects of High-Intensity Interval Training on Steroid Hormones and Psychological Outcomes in Healthy Male Adolescents: A Randomized Controlled Trial

**DOI:** 10.3390/sports14050209

**Published:** 2026-05-19

**Authors:** Nejmeddine Ouerghi, Wissal Abassi, Nidhal Jebabli, Mohamed Bessem Hammami, Anissa Bouassida, Katja Weiss, Thomas Rosemann, Moncef Feki, Beat Knechtle

**Affiliations:** 1 Research Unit “Sport Sciences, Health and Movement” (UR22JS01), High Institute of Sport and Physical Education of Kef, University of Jendouba, EI Kef 7100, Tunisia; najm_ouerghi@hotmail.com (N.O.); wissalabassi93@gmail.com (W.A.); jnidhal@gmail.com (N.J.); bouassida_anissa@yahoo.fr (A.B.); 2 LR99ES11, Faculty of Medicine of Tunis, Rabta Hospital, University of Tunis El Manar, Tunis 1007, Tunisia; bessem_hammami@yahoo.fr (M.B.H.); monssef.feki@gmail.com (M.F.); 3 Institute of Primary Care, University of Zürich, 8091 Zürich, Switzerland; katja@weiss.co.com (K.W.); thomas.rosemann@usz.ch (T.R.); 4 Medbase St. Gallen Am Vadianplatz, 9000 St. Gallen, Switzerland

**Keywords:** adolescent, anxiety, cortisol, depression, interval training, stress, testosterone

## Abstract

The study investigated the effects of high-intensity interval training (HIIT) on cardiorespiratory fitness, hormonal, and psychological markers in adolescents. Twenty-eight healthy male adolescents were randomized to a HIIT group or a non-training control group. HIIT comprises three sessions per week for 10 weeks, alternating 30 s runs at high-intensity and low-intensity. VO_2max_ was estimated using the incremental running test. Plasma testosterone and cortisol were assessed by ELISA methods. Depression, anxiety, and stress scores were determined using the Depression Anxiety Stress Scales-21. Data were analyzed using two-way ANOVA with repeated measures. Significant “group × time” interactions were detected for VO_2max_, testosterone, cortisol, testosterone-to-cortisol ratio, and stress score, but not for anxiety and depression scores. HIIT resulted in increased VO_2max_ (*p* < 0.001, d = 1.04), testosterone (*p* = 0.005, d = 0.52), and testosterone-to-cortisol ratio (*p* = 0.008, d = 1.05), and decreased cortisol (*p* = 0.036, d = 1.09) and stress score (*p* = 0.020, d = 0.98). Ten-week HIIT resulted in an improvement in physical fitness, steroid hormonal balance, and self-reported stress symptoms, but no changes in depressive and anxiety symptoms in comparison to the control group. The findings should be interpreted with caution due to limitations, including the small sample size and the lack of assessment of sex-related differences. Further research is required to elucidate the topic.

## 1. Introduction

Adolescence corresponds to the transition from childhood to adulthood, during which individuals become sexually mature and capable of reproducing [1]. The period is marked by key physical, physiological, and psychological transformations, particularly metabolic and hormonal changes [1]. Physical activity plays a substantial role in enhancing the physical and mental health of children and adolescents [2]. For children and adolescents, physical exercise induces metabolic and hormonal adaptations that may influence maturation, metabolic and psychological states [2,3]. The steroid hormones testosterone and cortisol are among the numerous hormones involved in the critical period of adolescence. Testosterone controls sexual maturation and exerts potent anabolic effects, promoting muscle development, strength, power, and endurance [4]. During puberty, testosterone production increases substantially in boys, influencing adolescents’ mood, self-esteem, and behavior, impacting mental health [5]. Cortisol acts as a catabolic hormone that mobilizes the main energy substrates (glucose, lipids, and proteins) necessary for muscle activity during physical exercise [6]. It also plays a crucial role in controlling physical and mental stress [7]. These hormonal parameters are essential for growth, metabolic regulation, recovery, and adaptation to exercise [8,9].

Adolescence is associated with a reduction in regular physical activity. Globally, less than 20% of adolescents accomplish the recommended levels of physical activity [10]. According to the 2020 WHO guidelines, children and adolescents should accumulate at least an average of 60 min per day of moderate-to-vigorous physical activity, primarily aerobic exercises, including vigorous-intensity and muscle- and bone-strengthening activities at least three times per week [11]. The decline in physical activity during adolescence affects cardiometabolic health and may contribute to mental disorders such as anxiety and depression [12]. Among the various modalities of physical activity, high-intensity interval training (HIIT) has emerged as a particularly effective approach, yielding significant benefits for physical fitness, cardiometabolic, and mental health [2,13,14].

Studies examining the effects of chronic HIIT intervention on testosterone and cortisol have focused on athletes, often using short-duration training, and yielded inconsistent data [15,16,17,18,19,20,21]. For instance, Zinner et al. [16] reported a significant increase in testosterone levels but no significant changes in cortisol or testosterone-to-cortisol ratio after 2-week HIIT in adolescent male triathletes. In contrast, Herbert et al. [18] reported a significant increase in cortisol levels and a decrease in testosterone-to-cortisol ratio, with no change in testosterone levels, following 6 weeks of HIIT in endurance athletes. Another study reported a significant increase in testosterone, associated with a downward trend of cortisol, following 3-week HIIT in young male athletes [17]. Conversely, Sylta et al. [19] showed a significant decrease in testosterone and testosterone-to-cortisol ratio after four weeks of high-intensity training in well-trained cyclists, with no change in cortisol; however, these hormones rebounded to baseline values after 8 and 12 weeks. Taken together, these findings highlight the heterogeneity of hormonal responses to HIIT, which may depend on training duration and intensity, participant characteristics, study design, and the timing of biological sample collection. A recent systematic review and meta-analysis reported that acute HIIT induces transient increases in testosterone and cortisol levels, suggesting that immediate endocrine responses may differ from long-term adaptations following chronic exercise intervention [22]. Definitely, the effects of HIIT on these steroid hormones in adolescents are still poorly understood.

Recent meta-analyses investigated the effect of HIIT on mental health. Tao et al. [23] reported moderate improvements in depressive symptoms, whereas Gaia et al. [24] found no significant effect of HIIT on symptoms of depression and anxiety in healthy individuals. These contrasting findings emphasize the need for further research, particularly in specific populations such as adolescents. During puberty, sex-specific hormonal regulation introduces additional variability in hormonal responses. Hence, the current study focused exclusively on male adolescents to reduce biological heterogeneity and improve the interpretability of hormonal adaptations. We hypothesized that the HIIT program would improve testosterone and cortisol levels and reduce the symptoms of stress, anxiety, and depression in adolescents. The current study aimed to evaluate the effects of a 10-week HIIT program on testosterone and cortisol levels, and selected psychological outcomes in healthy male adolescents.

## 2. Materials and Methods

### 2.1. Study Design and Procedures

A randomized clinical trial was conducted during the academic year 2024–2025 involving healthy male adolescents. Participants were randomized to an HIIT group or a non-training group. Plasma cortisol and testosterone, cardiorespiratory fitness indices, and psychological measures were determined in participants before and after a ten-week intervention. Qualified specialists supervised HIIT sessions and performed physical, biochemical, and psychological evaluations.

### 2.2. Ethical Approval

This study was conducted in accordance with the latest version of the Declaration of Helsinki, and the paper was written in line with the CONSORT guidelines. The study protocol was approved by the local ethics committee of the High Institute of Sport and Physical Education of Kef (ISSEPK-058/2024). Participants’ parents or legal guardians gave written informed consent. The study protocol was registered at ClinicalTrials.gov under the identifier NCT07311460 on 31 December 2025.

### 2.3. Sample Size Calculation

The sample size calculation was conducted using G*Power software (version 3.1.9.4, Heinrich-Heine-Universität Düsseldorf, Düsseldorf, Germany). Based on findings from a previous related study [25] examining the effects of HIIT on primary outcome MAS in adolescents, a priori power analysis was performed with a type I error rate (α) of 0.05 and a statistical power of 80%. The analysis indicated that 28 participants would be required to detect significant interaction effects for MAS (Cohen’s f = 0.55).

### 2.4. Participants

**Figure 1:**
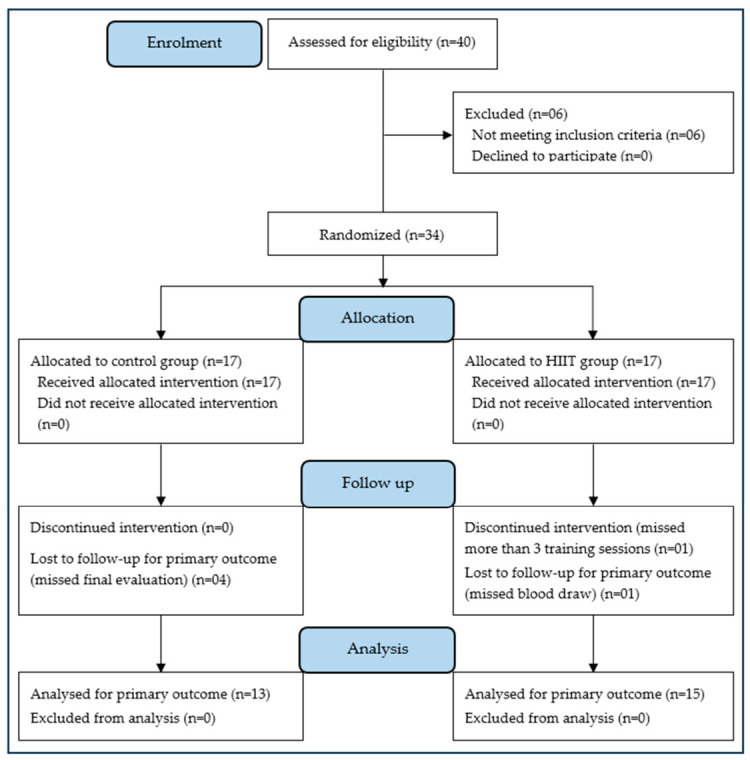
Flow chart of the study participants. HIIT, high-intensity interval training.

A total of 40 healthy adolescent males volunteered to participate in the study. Recruitment was conducted in public middle schools in Dahmani (Tunisia). Six of the volunteers did not meet the eligibility criteria. The remaining 34 adolescents were randomly assigned to the HIIT group (HIITG, *n* = 17) or the non-training control group (CG, *n* = 17). Inclusion criteria were male sex, age 14 to 16 years, normal weight according to the WHO child growth standards for body mass index (BMI) (BMI ≤ 85th percentile), and parental approval. Non-inclusion criteria were medical conditions contraindicating intense physical exercise, habitual physical activity exceeding school physical education lessons, participation in a physical training program within the past 6 months, and current or recent (within 3 months) dietary supplementation or restriction. Exclusion criteria were withdrawal of consent, non-compliance with the training program, or incomplete data. Randomization was conducted using a computer-generated list created with Microsoft Excel, ensuring a 1:1 allocation ratio. The randomization sequence was generated by an independent researcher who was not involved in the enrollment, assessment, or training of participants. Group assignments were concealed in sealed, opaque, and sequentially numbered envelopes to maintain allocation concealment. Envelopes were opened only after baseline measurements were completed. Due to the nature of the intervention, neither participants nor trainers were blinded to the group allocation. However, outcome assessors and data analysts were blinded to group assignments to minimize bias. Of the 34 participants, six dropped out of the training program for personal reasons (two from the HIITG and four from the CG). Finally, 28 male adolescents (age, 14.9 ± 0.74 yrs; height, 1.66 ± 0.07 m; BMI, 18.2 ± 2.67 kg/m^2^) completed the study: 15 in the HIITG and 13 in the CG (Figure 1). All participants underwent a comprehensive medical examination, which included anthropometric measurements and an assessment of genital maturation. Participants’ physical activity consisted of two-hour physical education classes, weekly. Habitual physical activity, eating behavior, and daily routines were maintained over the intervention period in all participants. Neither group received additional care or interventions during the study. According to the Tanner classification [26], all participants were pubescent at the time of inclusion. To ensure confidentiality, all participant data were anonymized before data analysis.

### 2.5. Aerobic Performance Measurement

Participants completed a graded exercise test (20 m shuttle run test) until exhaustion to assess cardiorespiratory fitness (CRF). Maximal aerobic speed (MAS) and maximal oxygen consumption (VO_2max_) were estimated using the incremental running test (20 m shuttle run test) as previously described [27]. The test was designed to assess the aerobic capacity of schoolchildren, healthy adolescents, and adults. The reproducibility of tests was determined using pilot data from 28 participants collected during familiarization trials [intra-class correlation coefficient (95% confidence interval) = 0.837 (0.647–0.924) for MAS and 0.850 (0.676–0.931) for VO_2max_].

### 2.6. High-Intensity Interval Training Intervention

The HIIT protocol consisted of three series of 30 s runs at 100–110% of MAS, and 30 s of active recovery at 50% of MAS. The training protocol was carried out as previously described [28,29] in young males. Table 1 provides a detailed overview of the training progression, including variations in the number of repetitions per set and exercise intensity. MAS was assessed before and after the intervention period. The reduction in training load during the program’s final phase was implemented as a tapering strategy to reduce accumulated fatigue while maintaining training-induced adaptations, optimizing performance. Based on previous HIIT studies in young people, total training workload was calculated as arbitrary training units (ATU) [28] as follows: ATU = [(work intensity + rest intensity)/2 × (number of repetitions × number of sets)] (Table 1). During the 10-week intervention, participant adherence was carefully monitored. In the HIIT group, 15 of 17 participants completed all scheduled training sessions, whereas one discontinued the intervention after missing more than three sessions, and another did not complete the final blood sampling. In CG, 13 of 17 participants completed the final evaluation, whereas four were lost to follow-up due to missing the final assessment (Figure 1). Participants who completed the intervention demonstrated total adherence to the program and evaluations. During the intervention period, no injuries or adverse events were reported.

### 2.7. Psychological Evaluations

We used the short form of the Depression Anxiety Stress Scales (DASS-21) [30]. The Scales are a 21-item self-report measure designed to assess the severity of general psychological distress and symptoms related to depression, anxiety, and stress in older adolescents. It assesses the frequency of behaviors or the intensity of feelings using four response options, ranging from 0 (does not apply to me at all) to 3 (applies to me a lot or most of the time). For each participant, raw subscale scores were used for all analyses.

### 2.8. Blood Sampling and Laboratory Analysis

Fasting blood samples were collected from the antecubital vein in the morning (around 8 a.m.) one day before the start and two days after the training completion. The samples were then centrifuged, and the plasma was frozen at −80 °C until analysis. Testosterone and cortisol concentrations were analyzed using enzyme-linked immunosorbent assay (ELISA) kits (Monobind, Inc., Lake Forest, CA, USA). The intra- and inter-assay coefficients of variation (CVs) were <6% and <12%, respectively.

### 2.9. Statistical Analysis

Data were expressed as means and standard deviations (SD). The normality of data in each group was checked using the Shapiro–Wilk test, and the homogeneity of variance was assessed using the Levene test. Two-way analyses of variance with repeated measures (ANOVA) were performed to test group-by-time interactions for physical, hormonal, and psychological variables. The effect size was estimated using partial eta squared (η_p_^2^), with values of 0.01, 0.06, and 0.14 or higher indicating small, medium, and large effect sizes, respectively. When a significant interaction effect was observed, Bonferroni-adjusted post hoc multiple comparisons were performed. For two-group comparisons, the effect size was calculated as Cohen’s d, which is considered small, moderate, and large for a value of 0.2 to 0.5, 0.5 to 0.8, and ≥0.8, respectively [31]. Changes in all variables, expressed as raw (post-test − pre-test), or percentage [(post-test − pre-test)/pre-test] × 100, were calculated and compared using independent *t*-tests. A per-protocol (complete-case) analysis was conducted, including only participants who completed the pre- and post-intervention assessments. Missing data were not imputed, and an intention-to-treat analysis was not performed. Statistical analyses were performed using the SPSS software version 22.0 (SPSS, Inc., Chicago, IL, USA). The level of statistical significance was set at *p* ≤ 0.05.

## 3. Results

Baseline anthropometric, aerobic fitness, hormonal, and psychological variables were not different between the HIIT and control groups. Table 2 summarizes pre- and post-intervention data, the “time × group” interaction, and the time and group effects for aerobic fitness variables, hormone concentrations, and psychological scores.

### 3.1. Cardiorespiratory Fitness

Estimated VO_2max_ and MAS demonstrated a significant “time × group” interaction. Intra-group analysis showed increases in MAS (*p* < 0.001, d = 1.15) and estimated VO_2max_ (*p* < 0.001, d = 1.04) after a 10-week intervention in the HIITG, but no significant changes in CG. Between-group analyses demonstrated higher post-intervention MAS (*p* = 0.045, d = 1.07) and estimated VO_2max_ (*p* = 0.028, d = 0.92) in the HIITG compared to the CG (Table 2).

### 3.2. Hormonal Parameters

**Figure 2:**
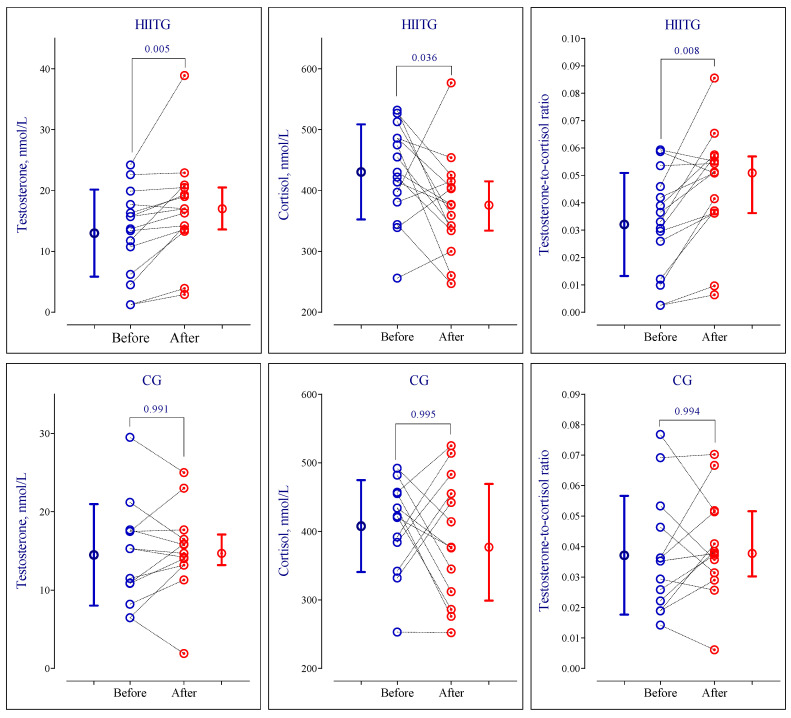
Changes in plasma testosterone and cortisol concentrations and the testosterone-to-cortisol ratio following a 10-week intervention program in high-intensity interval training (HIITG) and control (CG) groups. Before, before the intervention program; after, after the intervention program.

A significant “time × group” interaction was detected for testosterone and cortisol concentrations as well as the testosterone-to-cortisol ratio. Intra-group analyses revealed a significant increase in testosterone (*p* = 0.005, d = 0.52) and testosterone-to-cortisol ratio (*p* = 0.008, d = 1.05), but a decrease in cortisol (*p* = 0.036, d = 1.09) after 10-week intervention in the HIITG (Table 2, Figure 2). The mean percentage changes were greater in the HIITG than CG for testosterone [mean (95%CI), +61.5% (+19.5%, +10.3%) vs. +10.3% (−14.9%, +35.6%); *p* = 0.040], and testosterone-to-cortisol ratio [+93.0% (+35.5%, +151%) vs. +19.5% (−12.7%, +51.8%); *p* = 0.030]. However, no difference was found for cortisol [−10.7% (−24.1%, +2.72%) vs. −2.34% (−18.6%, +13.9%); *p* = 0.395]. No significant changes were observed for all hormonal measures in the CG.

### 3.3. Mental Health Measures

**Figure 3:**
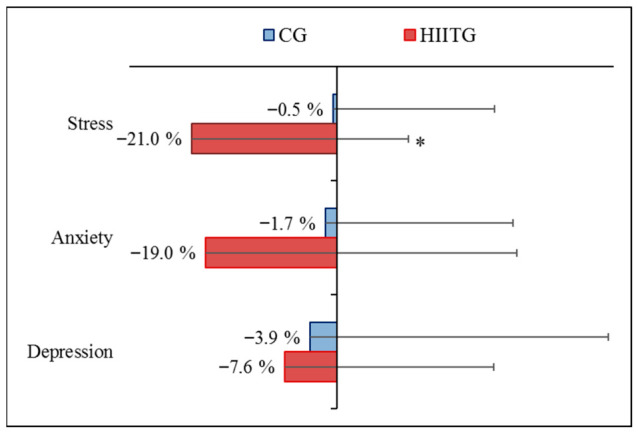
Mean percentage changes in depression, anxiety, and stress scores following a 10-week intervention in high-intensity interval training (HIITG) and control (CG) groups. *, *p* < 0.05 (compared to pre-intervention program).

A significant main effect of time was found for stress (*p* = 0.002) and anxiety (*p* = 0.020) scores. A significant “time × group” interaction was observed for stress (*p* = 0.045), but not for anxiety or depression scores. HIIT resulted in significant decreases in stress (*p* = 0.020, d = 0.98) and anxiety (*p* = 0.010, d = 0.79), but not in depression scores. No significant changes were found in the CG (Table 2). The mean percentage changes were greater in the HIITG than CG for stress score [mean (95% CI), −21.8% (−34.7%, −8.90%) vs. −0.33% (−19.3%, +18.7%); *p* = 0.048]. However, no differences were found for anxiety [−19.8% (−34.8%, −4.79%) vs. −1.67% (−28.9%, +25.5%); *p* = 0.201], and depression [−7.65% (−31.4%, +16.3%) vs. −3.85% (−22.1%, +14.4%); *p* = 0.798] scores (Figure 3).

### 3.4. Between-Group Changes in Aerobic Fitness, Hormonal, and Psychological Variables

Comparison of changes in variables between the HIIT and control groups revealed significant differences for MAS, VO_2max_, testosterone concentrations, and stress score. In contrast, no significant differences were found for changes in cortisol concentrations, testosterone-to-cortisol ratio, and depression and anxiety scores (Table 3).

## 4. Discussion

A ten-week HIIT program improved CRF, increased testosterone levels and testosterone-to-cortisol ratio, and decreased cortisol levels. The training also resulted in a decrease in self-reported symptoms of stress, but no changes in anxiety and depression symptoms. The findings suggest that HIIT is a time-efficient, non-pharmacological intervention that ameliorates steroid hormone balance and psychological outcomes in male adolescents. The current study confirms the beneficial effect of HIIT on CRF in adolescents and youths [14,32]. CRF is a key indicator of health and a predictor of cardiovascular risk [33]. García-Hermoso et al. [34] stated that early interventions targeting CRF in children may help maintain health parameters later in life. The improvement in CRF is explained by central and peripheral adaptations, such as increased cardiac output, hemoglobin levels, muscle oxidative capacity, and mitochondrial enzyme activities [35,36].

Studies that examined the response of steroid hormones to HIIT have yielded inconsistent results. HIIT programs often increased testosterone [15,16,17,20,21] but also resulted in no change [19] or a decrease [18] in the hormone levels. Cortisol has often remained unchanged [15,16,19] or decreased [20,21], but has also increased following HIIT programs. Discrepancies are likely due to differences in participants’ characteristics (e.g., age, development stage for adolescents, body composition, level of physical aptitude, psychological status, baseline hormonal levels), the duration, intensity, and modalities of HIIT programs, and the timing of blood sampling. These variations highlight the need for further research on hormonal adaptations to this type of training, particularly in adolescents, given the scarcity of data in this population. Most studies have shown an increase in testosterone under HIIT, which may be related to central and peripheral metabolic regulations.

While improving blood flow and brain oxygenation and inducing the release of endorphins, intensive physical exercise activates the hypothalamic–pituitary–gonadal axis [37], which stimulates testosterone synthesis/secretion by Leydig cells. The exercise also increases local blood flow, increasing the supply of oxygen and nutrients to the testicles and synthesis capacity. The training enhances muscle mass, increasing metabolic needs and shifting towards anabolic hormonal regulation, including stimulation of testosterone synthesis [15,38]. HIIT reduces fat mass and adipose tissue-associated aromatase activity, thus limiting the conversion of testosterone to estradiol [39]. While reducing psychological stress, the training may decrease cortisol secretion, which stimulates testosterone synthesis [40].

Cortisol concentration has been proposed as a criterion for adrenal cortex response to maximal physical exertion [41]. The HIIT program resulted in a significant decrease in cortisol levels, which may reflect a possible adaptation and improved resilience to physical stress induced by the exerting training. The testosterone-to-cortisol ratio has been used to monitor the balance between anabolic and catabolic activities and to track training load and physiological strain [42]. The increase in the ratio in these adolescents reflects beneficial metabolic, physiological, and psychological adaptations to HIIT. Romero-Martínez et al. [43] demonstrated that a high ratio is associated with better self-esteem and improved mental health in healthy individuals.

Physical activity is believed to enhance mental health. A systematic review showed that all types of physical activity are effective, and higher intensity exercise is linked to greater improvements in depression, anxiety, and psychological distress across various adult groups, including the general population and patients with mental health disorders or chronic diseases [44]. A meta-review of systematic reviews suggests that HIIT improves anxiety and depression throughout the lifespan [32]. Most research on the topic has focused on adult and older adult populations. However, the effects of HIIT on mental health in adolescents are not firmly established. A review by Leahy et al. [2] suggests that HIIT may improve cognitive function and mental health in children and adolescents. However, due to the small number and large heterogeneity of included studies, further research is necessary to settle the results. Costigan et al. [45] found no significant effect of an 8-week HIIT program on depression and anxiety in healthy adolescents. In the current study, a 10-week HIIT program reduced self-reported symptoms of stress and anxiety. These findings should be interpreted with caution due to potential floor effects, given the low baseline scores in both groups. Furthermore, in a context where the participants could not be blinded to the group, and the control group did not receive an attention-matched intervention, expectancy or contextual effects may have been introduced. The positive impact may be partly due to the secretion of neuroendocrine factors, such as endorphins, the modulation of the autonomic nervous system, the control of inflammation, and improvements in CRF, all of which are associated with positive mood and well-being [46]. However, these mechanisms remain speculative. On the other hand, engaging in challenging physical activities, such as HIIT, enhances adolescents’ sense of self-efficacy, boosts their confidence, and prepares them to better deal with stressors.

The HIIT program resulted in no significant change in depression scores. Lack of effect may be partly explained by the low baseline levels of depressive symptoms, which could limit the ability to detect significant improvements. Furthermore, depressive symptoms were assessed using self-reporting scales that may lack sensitivity in detecting subtle changes, particularly in non-clinical populations. The absence of changes in the current study is consistent with the literature data [2,23,24], which reflects differences in baseline psychological status, participant characteristics, and intervention protocols. Overall, the impact of HIIT on depressive symptoms in healthy adolescents may be limited or context dependent. Future studies should involve more sensitive, multidimensional assessments of depressive symptoms.

The study has limitations that should be acknowledged. Firstly, it involved male adolescents, which limits its applicability to adolescents of both sexes. Due to sex-specific differences in hormonal status and its potential impact on psychology during adolescence, examining hormonal and psychological responses to HIIT in female adolescents is needed. Dietary intake, sleep, and circadian rhythm, which could influence both hormonal and psychological outcomes, were not controlled. Yet, all participants were instructed to maintain their usual eating habits and daily routines throughout the intervention period. The lack of an active comparator group—i.e., participants performing moderate-intensity continuous training—limits the ability to isolate the specific effects of HIIT. Some of the improvements may reflect the general introduction of structured physical activity rather than the intensity or modality of intervention itself. The lack of objective monitoring of exercise intensity (e.g., heart rate, accelerometry) might limit the reproducibility and internal validity. Future research should integrate objective tools to enhance the quantification of exercise load. Furthermore, cardiorespiratory fitness was assessed using a field-based VO_2max_ estimate rather than direct laboratory measurement, which may reduce the measurement precision. The hormonal outcomes were based on single fasting morning samples, which are subject to biological variability in adolescents. The psychological outcomes were assessed using the self-reported questionnaire, which may introduce reporting bias. The maturation status was not included as a covariate in the statistical analyses, which may represent a limitation, as variation in pubertal development could influence physical, hormonal, and psychological outcomes. However, this is unlikely as most participants were at Tanner stage 4, with no differences between the two groups by the Tanner stage. The relatively small sample size may have limited statistical power. Finally, because of limitations, the study findings should be interpreted with caution. Future studies should overcome these limitations.

## 5. Conclusions

Ten weeks of HIIT significantly improved CRF, increased testosterone and the testosterone-to-cortisol ratio, and decreased cortisol and stress in healthy male adolescents. The anxiety score has decreased only in the HIIT group. There were no significant variations in depression scores between the HIIT and control groups. These changes suggest an anabolic-type hormonal adaptation. The study findings support that HIIT may be effective in enhancing physical fitness, hormonal status, and psychological outcomes in adolescents. Further research is needed to test the long-term effects of HIIT or other physical training on hormonal and psychological outcomes, alone or in combination with nutritional programs, among healthy and clinical adolescent populations. The mechanisms underlying the relationship between HIIT-associated hormonal, physical, and psychological changes require further investigation.

## Figures and Tables

**Table 1:** Summary of the high-intensity interval training (HIIT).

	Week of Training				
	1–2	3–4	5–6	7–8	9–10
Number of sets	3	3	3	3	3
Number of races per set	4	6	6	8	6
Run/Active recovery time (s)	30/30	30/30	30/30	30/30	30/30
% MAS (Run/Active recovery)	100%/50%	100%/50%	110%/50%	100%/50%	100%/50%
Passive recovery time (min)	5	5	5	5	5
Training load (ATU)	900	1350	1440	1800	1350
Weekly training load (ATU)	2700	4050	4320	5400	4050

MAS, maximal aerobic speed; ATU, arbitrary training unit; s, second. Example: For the first two weeks, the participant completes three sets of four repetitions of 30 s running at 100% MAS, and 30 s of active recovery at 50% MAS. The subject recovers passively for 5 min between each set. Training load is calculated as follows: (100 + 50)/2 × (3 × 4 ATU).

**Table 2:** Aerobic, hormonal, and psychological measures and “time × group” interactions following a 10-week high-intensity interval training (HIIT) program.

	Control Group (*n* = 13)		HIIT Group (*n* = 15)		*p* ^a^	Time Effect ^b^		Group Effect ^b^		Interaction (Time × Group) ^b^	
	Pre	Post	Pre	Post		F	η_p_^2^	F	η_p_^2^	F	η_p_^2^
MAS (km/h)	11.1 ± 0.55	11.2 ± 0.72	11.2 ± 0.62	11.9 ± 0.64 ***^†^	0.707	33.5 ^###^	0.563	1.87	0.067	6.45 ^#^	0.199
VO_2max_ (mL/kg/min)	43.6 ± 3.40	44.0 ± 4.03	43.7 ± 3.65	47.4 ± 3.70 ***^†^	0.926	28.9 ^###^	0.527	1.02	0.038	11.0 ^##^	0.298
Testosterone, nmol/L	14.5 ± 6.48	15.1 ± 5.54	13.0 ± 7.15	16.9 ± 8.29 **	0.567	14.6 ^##^	0.359	0.78	0.029	8.74 ^##^	0.252
Cortisol, nmol/L	408 ± 67.0	389 ± 91.8	431 ± 78.1	374 ± 81.4 *	0.417	18.5 ^###^	0.415	0.14	0.005	5.92 ^#^	0.185
T/C ratio	0.039 ± 0.02	0.042 ± 0.01	0.032 ± 0.01	0.048 ± 0.02 **	0.402	14.8 ^###^	0.363	0.34	0.013	5.21 ^#^	0.167
Depression score	3.85 ± 1.21	3.54 ± 1.05	3.93 ± 1.03	3.40 ± 1.18	0.839	4.89 ^#^	0.158	0.010	0.004	1.02	0.038
Anxiety score	2.92 ± 1.12	2.62 ± 0.87	3.13 ± 1.06	2.40 ± 0.83 **	0.614	12.4 ^##^	0.323	0.29	0.011	4.12	0.137
Stress score	4.85 ± 1.95	4.54 ± 1.66	5.07 ± 1.58	3.80 ± 1.21 *	0.744	13.8 ^##^	0.347	0.11	0.004	5.78 ^#^	0.182

Data are expressed as mean ± SD; Pre, before the intervention; Post, at the end of the intervention; MAS, maximal aerobic speed; VO_2max_, maximal oxygen consumption; η_p_^2^, partial eta squared; T/C, testosterone-to-cortisol ratio. *, *p* < 0.05; **, *p* < 0.01; ***, *p* < 0.001 (intra-group comparison by bonferoni-adjusted two-way repeated measures ANOVA); ^†^, *p* < 0.05; (between-group comparison by bonferoni-adjusted two-way repeated measures ANOVA); ^a^, baseline comparisons using independent *t*-tests; ^b^, interaction (time × group), as well as time and group effects were tested using bonferoni-adjusted two-way repeated measures ANOVA; ^#^, *p* < 0.05; ^##^, *p* < 0.01; ^###^, *p* < 0.001.

**Table 3:** Comparative changes (post-test − pre-test) in aerobic fitness, hormonal, and psychological variables in the high-intensity interval training group and control group.

	Mean (95% Confidence Interval)		*p*-Value
	Control Group	HIIT Group	
MAS (km/h)	0.08 (−0.17, +0.32)	0.67 (+0.50, +0.84)	<0.001
VO_2max_ (mL/kg/min)	0.05 (−0.89, +1.80)	3.77 (+2.78, +4.75)	<0.001
Testosterone, nmol/L	0.62 (−1.77, +3.00)	3.94 (+1.55, +6.33)	0.044
Cortisol, nmol/L	−18.6 (−84.7, +47.5)	−56.6 (−114, +1.26)	0.357
Testosterone-to-cortisol ratio	0.003 (−0.007, +0.013)	0.015 (+0.006, +0.023)	0.075
Depression score	−0.31 (−0.88, +0.26)	−0.53 (−0.34, +0.27)	0.637
Anxiety score	−0.31 (−1.06, +0.45)	−0.73 (−1.27, −0.20)	0.318
Stress score	−0.31 (−1.02, +0.41)	−1.27 (−1.94, −0.59)	0.045

MAS, maximal aerobic speed; VO_2max_, maximal oxygen consumption.

## Data Availability

The data presented in this study are available upon request from the corresponding author for privacy reasons.

## Abbreviations

The following abbreviations are used in this manuscript:

BMI	Body mass index
CRF	Cardiorespiratory fitness
CG	Control group
DASS-21	Depression Anxiety Stress Scales
HIIT	High-intensity interval training
HIITG	High-intensity interval training group
MAS	Maximal aerobic speed
VO_2max_	Maximal oxygen consumption
